# HSPB1 deficiency sensitizes melanoma cells to hyperthermia induced cell death

**DOI:** 10.18632/oncotarget.11894

**Published:** 2016-09-08

**Authors:** He-Xiao Wang, Yang Yang, Hao Guo, Dian-Dong Hou, Song Zheng, Yu-Xiao Hong, Yun-Fei Cai, Wei Huo, Rui-Qun Qi, Li Zhang, Hong-Duo Chen, Xing-Hua Gao

**Affiliations:** ^1^ Department of Dermatology, The First Hospital of China Medical University, Shenyang 110001, P.R. China

**Keywords:** hyperthermia, thermotolerance, HSPB1, combination strategy, melanoma

## Abstract

Hyperthermia has shown clinical potency as a single agent or as adjuvant to other therapies in cancer treatment. However, thermotolerance induced by thermosensitive genes such as the heat shock proteins can limit the efficacy of hyperthermic treatment. In the present study, we identified HSPB1 (HSP27) is hyperthermically inducible or endogenously highly expressed in both murine and human melanoma cell lines. We used a siRNA strategy to reduce HSPB1 levels and showed increased intolerance to hyperthermia via reduced cell viability and/or proliferation of cells. In the investigation of underlying mechanisms, we found knock down of HSPB1 further increased the proportion of apoptotic cells in hyperthermic treated melanoma cells when compared with either single agent alone, and both agents leaded to cell cycle arrest at G0/G1 or G2/M phases. We concluded that hyperthermia combined with silencing of HSPB1 enhanced cell death and resulted in failure to thrive in melanoma cell lines, implying the potential clinical utility of hyperthermia in combination with HSPB1 inhibition in cancer treatment.

## INTRODUCTION

Hyperthermia is a procedure by which the temperature of a specific body part or the whole organism is elevated above the standard physiological temperature [1]. A temperature range of 42°C to 45°C is used therapeutically as a single agent or as an adjuvant to radiotherapy, chemotherapy or immunotherapy in the cancer treatment [2, 3]. Hyperthermia alone as a cancer treatment can selectively eliminate cancer cells by facilitating molecular mechanisms such as cell cycle arrest, apoptosis, necrosis and autophagy [4–6], or activate NK cells or DCs which in turn stimulates anti-cancer immune responses [7, 8]. Both *in vitro* and *in vivo* studies have reported a clinical benefit from use of hyperthermia as a treatment for many cancers including melanoma [5, 9, 10], prostate cancer [11], bladder cancer [12] and glioblastoma [13]. Hyperthermia acts as a sensitizer to radiotherapy, chemotherapy and immunotherapy, and thus, this has attracted interest in developing effective combination strategies that exploit using hyperthermia in combination with other therapies. Successful combinations involving hyperthermia have been reported in breast cancer [14], bladder cancer [15, 16], cervical cancer [17] and prostate cancer [18]. Therefore there is interest in developing effective dual therapies that exploit the use of hyperthermia.

Hyperthermia regulates a family of molecular chaperone proteins, the heat shock proteins (HSPs) [19]. HSPs are highly conserved and constitutively expressed [20]. They function to facilitate the folding, conformation, assembly, and translocation of proteins involved in cell growth and survival. Therefore, they have important roles in human diseases including cancer [21, 22]. There is a precedence for heat shock proteins being associated with increased thermotolerance [23, 24]. HSP70 is perhaps the best studied in this regard, and HSP70 inhibitors have been shown to have anticancer effects [25–28]. However, the thermoregulatory role of HSP70 has the potential to be confused with its anti-immune activity [29–31]. Another heat shock protein, HSP27, is perhaps a better candidate. Also known as HSPB1, it is a small HSP that plays an essential role in the cytoprotection in cancer, and is inducible by various stimuli such as hyperthermia [32]. HSPB1 targets multiple components in the apoptosis signaling pathway to reduce levels of apoptosis [33]. When overexpressed in cancer HSPB1 is related to poor prognosis, tumour progression and metastasis [34–36]. All these features make HSPB1 an attractive therapeutic target, and indeed HSPB1 inhibitors have been revealed to be clinically effective in inhibiting tumour progression, promoting apoptosis and sensitizing cancer cells to other chemotherapies in pancreatic cancer, head and neck squamous cell carcinoma and prostate cancer [37–40].

The efficacy of hyperthermia can be limited by thermotolerance, which is a phenomenon in which cells become resistant to the heat treatment [2]. Hyperthermia induced HSPs may function to protect cells against hyperthermia activated cell death mechanisms such as necrosis, apoptosis and cell cycle arrest, and thus, may be responsible for this thermotolerance [24, 41]. Therefore, silencing thermosensitive HSPs may improve the antitumour effects of hyperthermia. Additionally, as a sensitizer to other therapies, hyperthermia may also enhance impaired cytoprotection attributed by HSP deficiency. In our study, we have shown HSPB1 is a thermosensitive HSP that was dramatically upregulated by hyperthermia of 45°C in the murine B16 melanoma cell line. Combination of HSPB1 silencing and hyperthermia significantly improved the impact of either treatment alone in terms of decreased cell viability, apoptosis and cell cycle arrest in B16 cells, as well as human cell lines with high HSPB1 expression, either endogenous or exogenously upregulated by hyperthermia, implying the potential clinical utility of hyperthermia in conjunction with HSPB1 silencing in melanoma treatment.

## RESULTS

### Hyperthermia (45°C) decreased the cell viability and upregulated Hspb1 expression in murine B16 melanoma cell line

**Figure 1:**
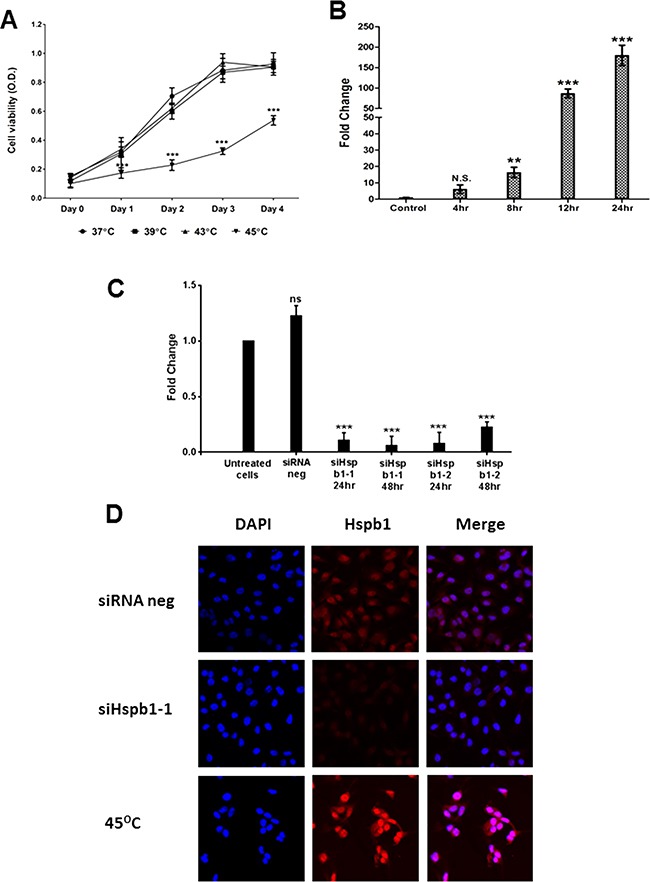
The cell viability and Hspb1 mRNA expression in murine B16 melanoma cell line after hyperthermic treatment, and knock down of Hspb1 by siRNA transfection **A.** MTS analysis showing the influence of cell viability of B16 cells by hyperthermia at 39°C, 43°C and 45°C, compared to 37°C (control group). Each point represents the mean ± SD of octuplicates. **B.** mRNA expression of Hspb1 in B16 cell line under hyperthermia time course, shown in means normalized against the endogenous control of B2m Data shown were means from three experiments ± SD. **C.** RNA was extracted from untreated B16 cells, cells treated with Silencer® Select negative control siRNA 1 (siRNA neg), and cells transfected by Mm_Hspb1_1 (siHspb1-1) or Mm_Hspb1_2 (siHspb1-2). The mRNA expression level of Hspb1 was normalized against the endogenous control of B2m. **D.** Representative confocal microscopy images showing Hspb1 expression in B16 cells treated with siRNA negative control, siHspb1-1 (48 hours after transfection) and hyperthermia (45°C). Hspb1 was labeled by TRITC (red) and nuclei were visualized by DAPI staining (blue).

We first measured the effect of hyperthermia on the cell viability of B16 cells by MTS assay. B16 cells were divided into four groups and treated with 37°C (negative control group), 39°C, 43°C and 45°C (hyperthermic treated groups) by water baths for 30 minutes, respectively. As shown in Figure 1A, there was no alteration in the cell viability of B16 cells under the conditions of 39°C or 43°C compared to that in the control group, but only in cells in the 45°C group which showed significantly reduced cell viability even after day 1 post heat shock (p<0.001). We also observed significantly induced upregulation of Hspb1 expression after hyperthermic treatment at 45°C (Figure 1B and 1D). There was a sustained increase in Hspb1 mRNA expression from 4 hours post hyperthermia application by RT-PCR analysis, peaking at 180 fold of increase at 24 hours (p<0.001).

Induction of HSPs overexpression is an essential signature of hyperthermia. We have identified that 45°C dramatically increased Hspb1 expression in B16 cells. To address if overexpressed Hspb1 influenced the thermosensitivity of B16 cells, Hspb1 was knocked down by siRNA transfection and cells were treated with a temperature change at 24 hours. As shown in Figure 1C, two Hspb1 targeted siRNA (siHspb1-1 and siHspb1-2) both effectively reduced up to more than 90% of Hspb1 mRNA expression after 24 hours of transfection (p<0.001). Hspb1 was still silenced in siHspb1-1 transfected cells after 48 hours, but started to re-express in siHspb1-2 transfected cells. Successful Hspb1 knock down was also proved at protein level by immunoflorescent staining (Figure 1D). siRNA transfected cells showed much lower level of Hspb1 expression compared with siRNA negative control cells. Despite the massive increase in Hspb1 expression in non-transfected 45°C treated B16 cells, Hspb1 expression in siHspb1-1 transfected 45°C treated B16 cells remains low (Supplementary Figure S1), indicating the successful silencing of Hspb1 by siHspb1-1 under both normal and hyperthermia conditions.

### Hspb1 knock down combined with hyperthermia (45°C) significantly reduced cell viability/proliferation compared with hyperthermia or Hspb1 knock down alone

**Figure 2:**
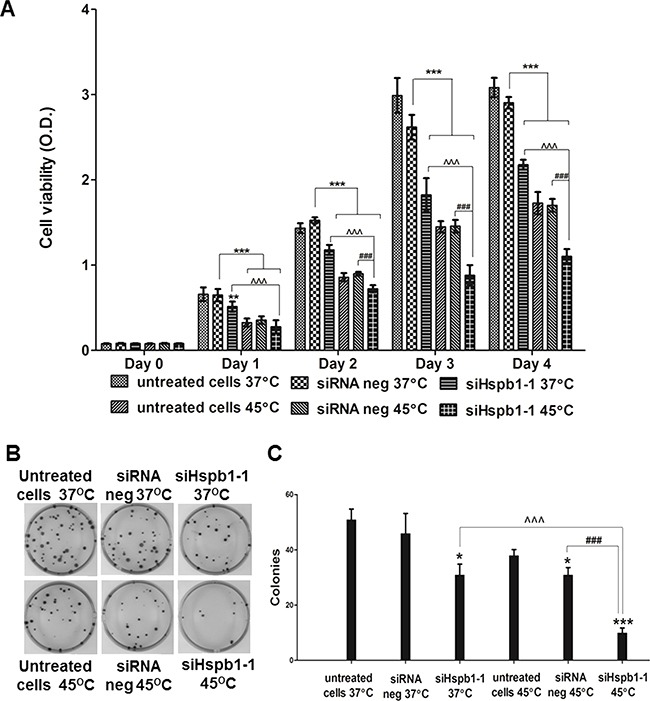
The effect of hyperthermia (45°C) and Hspb1 knock down on cell viability and colony formation of B16 cell line **A.** MTS analysis of B16 cell viability after heat shock (45°C) and Hspb1 knock down. **B.** Representative images of the colony formation assay. **C.** Quantification of the colony formation efficiency. * indicates a significant difference between each group and siRNA negative (37°C) (* p<0.05, ** p<0.01, *** p<0.001, by one way ANOVA); ^^^ indicates a significant difference between siHspb1-1 (45°C) and siHspb1-1 (37°C) (p<0.001, by student's t-test); ### indicates a significant difference between siHspb1-1 (45°C) and siRNA negative (45°C) (p<0.001, by student's t-test).

Having demonstrated that hyperthermia (45°C) reduced the cell viability of B16 cells, MTS assay was then used to compare the impact of hyperthermia (45°C), Hspb1 knock down, and the combination of both on B16 cell viability (Figure 2A). The same number of cells from untreated cells (incubated with medium), Silencer® Select negative control siRNA 1 treated cells (siRNA neg) and Mm_Hspb1_1 treated cells (siHspb1-1), were transferred into 96-well plates and measured by MTS assay on day 0. Each group was then divided into two subgroups and incubated with 37°C or 45°C, respectively. The cell viability was measured from the following day (day 1) until day 4. Compared to the siRNA negative (37°C), 45°C or Hspb1 knock down both significantly reduced the cell viability. There was a further reduction in the cell viability under the condition of a combination of both 45°C and Hspb1 knock down (p<0.001), suggesting the combination strategy enhanced single agent induced cell death. Colony formation assay also confirmed this finding since there was a significantly lower colony forming efficiency in the combination group compared to 45°C hyperthermia or Hspb1 knock down alone (Figure 2B and 2C).

### Regulation of apoptosis by hyperthermia (45°C) and Hspb1 knock down

**Figure 3:**
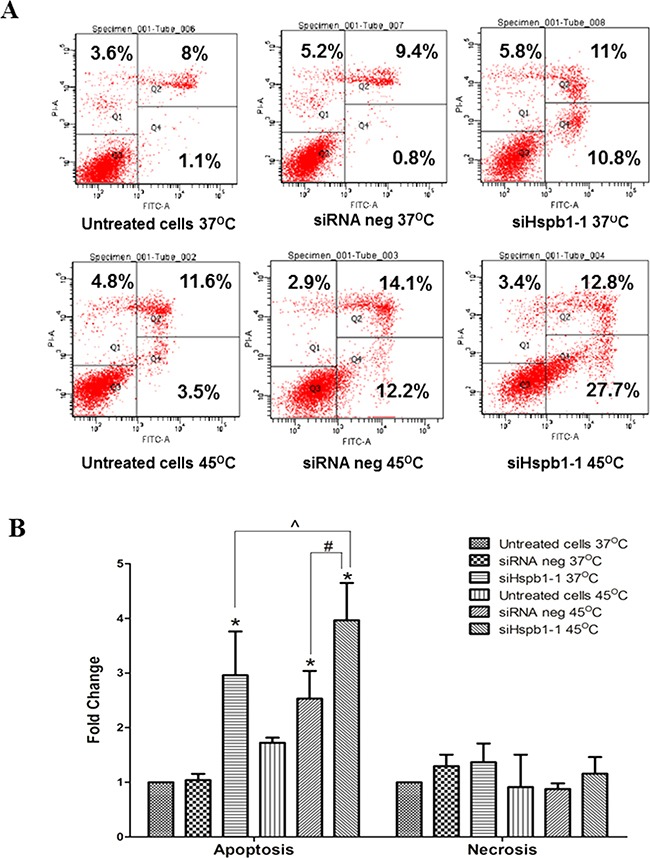
The effect of hyperthermia (45°C) and Hspb1 knock down on apoptosis and necrosis in B16 cell line analysed by flow cytometry **A.** Flow cytometric images of apoptosis and necrosis in B16 cells treated with hyperthermia and siHspb1-1 transfection. Cells stained with Annexin V+/ PI−, Annexin V+/ PI+, and Annexin-V−/PI+ were interpreted as early apoptotic cells, late apoptotic cells, and necrotic cells, respectively. **B.** Apoptosis and necrosis level of B16 cells were shown as fold change against siRNA negative group (37°C), and data shown were means from three independent experiments ± SD. * indicates a significant difference between each group and siRNA negative (37°C) (* p<0.05, by one way ANOVA); ^ siHspb1-1 (45°C) vs siHspb1-1 (37°C) (p<0.05, by student's t-test); # siHspb1-1 (45°C) vs siRNA negative (45°C) (p<0.05, by student's t-test).

Hyperthermia and Hspb1 are both involved in the regulation of apoptosis and necrosis. In order to address the possible mechanisms by which hyperthermia (45°C) combined with Hspb1 knock down reduces the cell viability and/or proliferation, we quantified the number of apoptotic cells by flow cytometry analysis (Figure 3). Compared to the siRNA negative group (37°C), there was a 1.7 and a 3.0 fold change of increase in the total apoptotic cells in the hyperthermia (45°C) group and Hspb1 knock down group, respectively (p<0.05) (Figure 3B). The total apoptotic cells further increased up to 4.0 fold change (p<0.05) in the combination group. However, there was no obvious difference in cell necrosis between each group. Taken together, 45°C treatment enhanced single agent induced cell apoptosis, but did not influence the cell necrosis.

### Knock down of Hspb1 induced G0/G1 cell cycle arrest, and hyperthermia (45°C) induced G2/M cell cycle arrest

**Figure 4:**
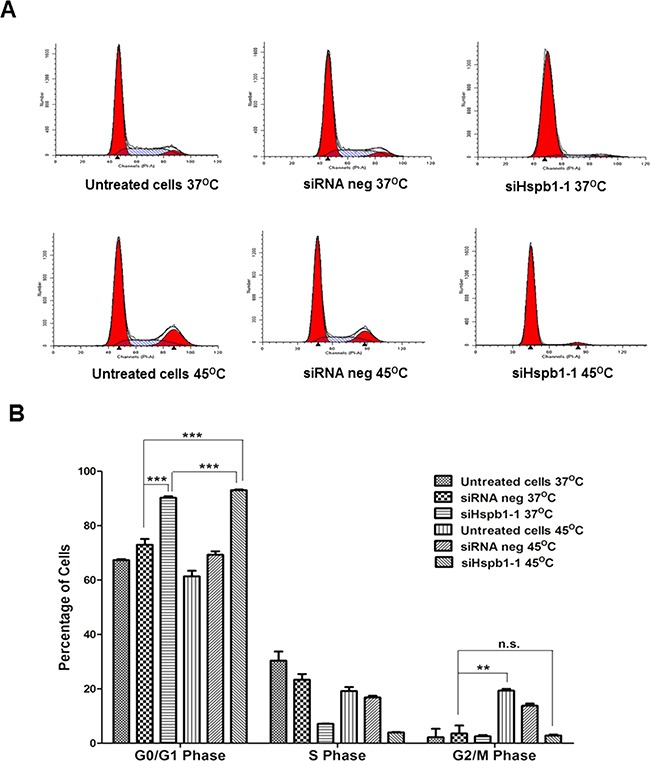
Flow cytometric analysis of the effect of heat shock (45°C) and Hspb1 siRNA transfection on cell cycle regulation in B16 cell line using PI staining **A.** Representative flow cytometric images of cell cycle distribution. **B.** Graphs showed the mean values from three independent experiments ± SD. Statistical significance was calculated by Student's t-test: NS, not significant, * p<0.05, ** p<0.01, *** p<0.001.

The colony formation results illustrated the impact of hyperthermia and Hsbp1 knock down on colony forming ability, which prompted us to investigate if cell cycle arrest was a mechanism involved in sensitizing B16 cells to hyperthermia (45°C) and Hspb1 knock down. As shown in Figure 4, the percentage of cells in the G2/M phase increased from 3.2% in the negative group (37°C) to 19.4% in the hyperthermia group, suggesting induction of G2/M cell cycle arrest by hyperthermia (p<0.001). Interestingly G2/M arrest did not occur when Hsbp1 siRNA treated cells were subjected to 45°C, suggesting G2/M arrest was not a mechanism of Hsbp1 action in hyperthermia. Hspb1 silencing, on the other hand, induced G0/G1 cell cycle arrest by showing an increase in the cell percentage from 73.0% (siRNA negative group, 37°C) to 90.3% (Hspb1 transfected group, 37°C) in this phase. Whereas in the combination treatment group, cells arrested in the G0/G1 phase were moderately increased to 93.1% when compared with Hspb1 knock down group (90.3%) (p<0.001). These results suggested a role of Hsbp1 as a powerful controller of cell cycle, and Hspb1 related cell cycle arrest may have gained additive effect by hyperthermic treatment.

### Hyperthermia (45°C) upregulated HSPB1 expression in human melanoma cell lines

**Figure 5:**
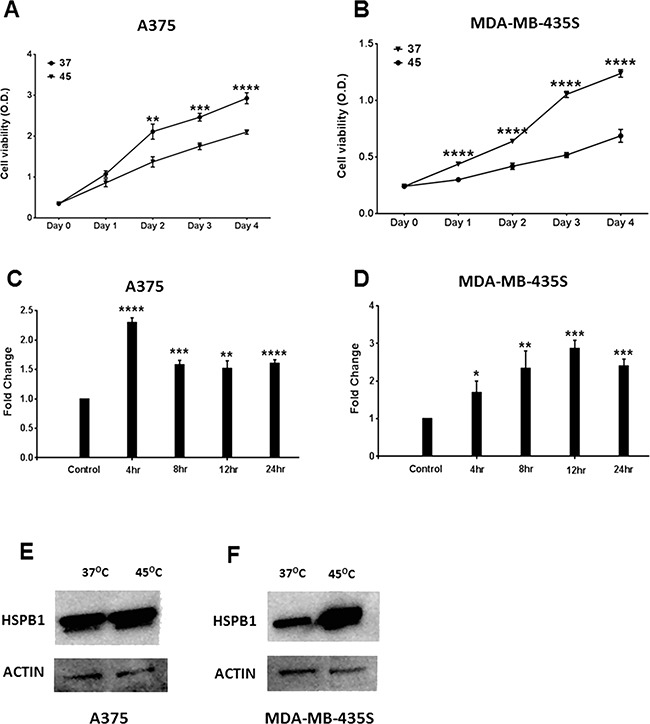
The cell viability and HSPB1 mRNA expression in human melanoma cell line after hyperthermic treatment **A, B.** MTS analysis showing the cell viability of A375 cells (A) and MDA-MB-435S cells (B) in control group (37°C) and hyperthermia group (45°C). Each point represents the mean ± SD of octuplicates. **C, D.** mRNA expression of HSPB1 in A375 and MDA-MB-435S cell lines under hyperthermia time course, shown in means normalized against the endogenous control of GAPDH. Data shown were means from three experiments ± SD. **E, F.** Western blots of HSPB1 protein expression in control group and hyperthermia group (45°C) in A375 and MDA-MB-435S cell lines. ACTIN was used as the loading control.

To further validate the findings from the murine B16 melanoma cell lines, we expanded our investigations into two human melanoma cell lines A375 and MDA-MB-435S. The thermo-sensitivity of these two lines was first measured by MTS assay (Figure 5A and 5B). Both cell lines showed significantly reduced cell viability upon hyperthermic stimulation (45°C) when compared to the control conditions (37°C), with MDA-MB-435S being more thermo-sensitive. The impact of hyperthermia on HSPB1 expression was then examined. HSPB1 mRNA expression level moderately increased in both A375 and MDA-MB-435S cell lines after hyperthermic treatment (Figure 5C and 5D). HSPB1 protein expression was abundantly increased in 45°C treated MDA-MB-435S cells (Figure 5F and Supplementary Figure S2B), but sustained at a high level in both 37°C and 45°C groups in A375 cells (Figure 5E and Supplementary Figure S2A).

### HSPB1 knock down combined with hyperthermia (45°C) significantly reduced cell viability/proliferation of human melanoma cell lines compared with hyperthermia or HSPB1 knock down alone

**Figure 6:**
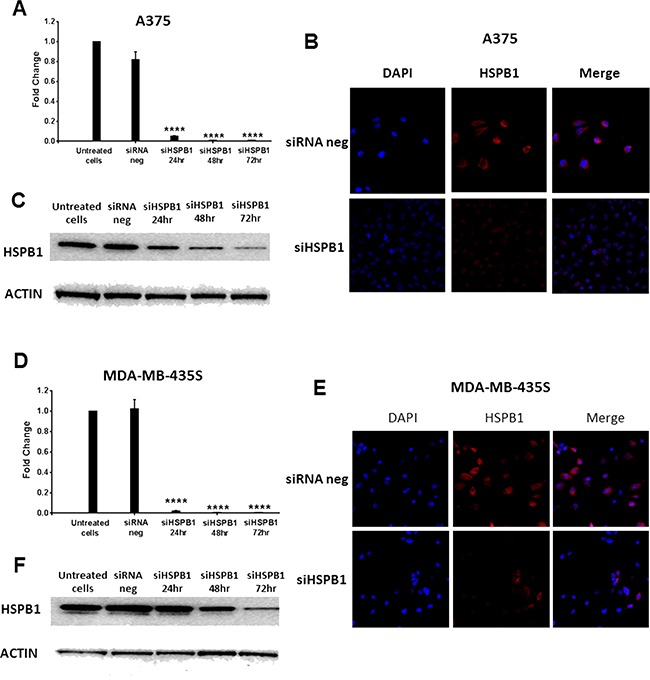
Knock down of HSPB1 by siRNA transfection in A375 and MDA-MB-435S cell lines **A, D.** RT-PCR analysis showing effective reduction of HSPB1 gene expression in A375 (A) and MDA-MB-435S (D) cell lines after 24 hours, 48 hours and 72 hours of transfection. The mRNA expression level of HSPB1 was normalized against the endogenous control of GAPDH. **B, E.**: Representative confocal microscopy images showing HSPB1 expression in two human melanoma cells treated with siRNA negative control and HSPB1 siRNA (after 48 hours of transfection). HSPB1 was labeled by TRITC (red) and nuclei were visualized by DAPI staining (blue). **C, F.**: Western blots showing reduced HSPB1 protein expression in A375 (C) and MDA-MB-435S (F) cell lines after 24 hours, 48 hours and 72 hours of transfection. Actin was used as the loading control.

**Figure 7:**
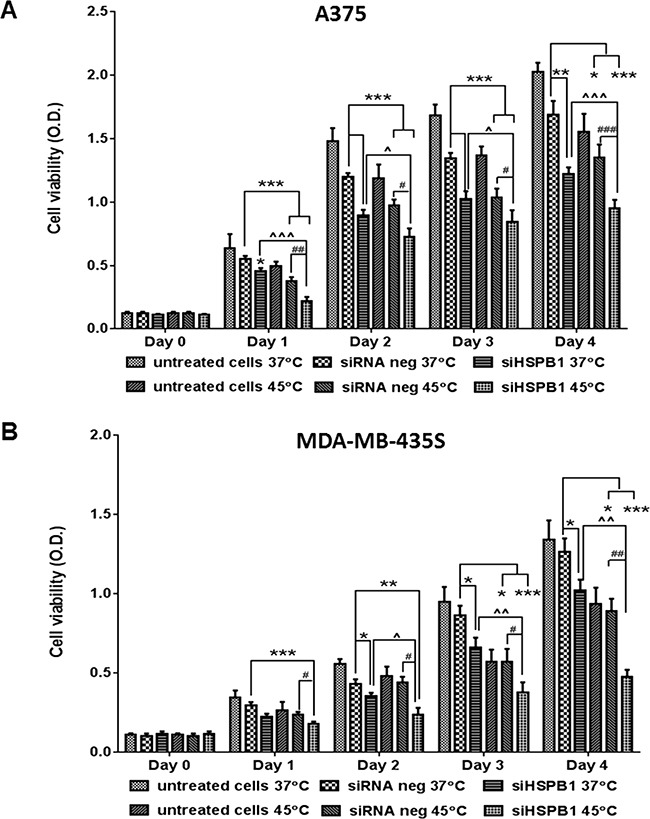
The effect of hyperthermia (45°C) and HSPB1 knock down on cell viability of A375 **A.** and MDA-MB-435S **B.** cell lines. * indicates a significant difference between each group and siRNA negative (37°C) (* p<0.05, ** p<0.01, *** p<0.001, by one way ANOVA); ^ indicates a significant difference between siHSPB1 (45°C) and siHSPB1 (37°C) (^ p<0.05, ^^ p<0.01, ^^^ p<0.001, by student's t-test); # indicates a significant difference between siHSPB1 (45°C) and siRNA negative (45°C) ((# p<0.05, ## p<0.01, ### p<0.001, by student's t-test).

HSPB1 was effectively knocked down in A375 and MDA-MB-435S cell lines by siRNA transfection at both mRNA and protein level (Figure 6A-6F). Consistent with our findings in B16 cells, we found that combining HSPB1 knockdown and hyperthermia produced a significantly larger reduction in cell viability than either agent alone: A375 (Figure 7A) and MDA-MB-435S (Figure 7B). Regardless of how the reduction of HSBP1 is achieved, melanoma cells without HSBP1 show increased thermosensitivity. Increase of apoptotic or necrotic cells (Supplementary Figure S3), and induction of cell cycle arrest at G0/G1 or G2/M phases (Supplementary Figure S4) were likely to be the essential underlying molecular mechanisms by which the combination strategy showed an additive effect over single agent treatment on reducing the cell viability.

## DISCUSSION

Here we present our data suggesting that silencing thermosensitive HSPB1 sensitized murine melanoma and human melanoma cell lines to hyperthermia induced cell death. It is known that HSPB1 expression increases dramatically upon hyperthermic insult and that reduction of HSPB1 activity with inhibitors will increase cancer cell sensitivity to a range of chemotherapies [32, 34–36]. Here using a siRNA strategy we reduced HSPB1 levels and showed increased intolerance to hyperthermia via reduced cell viability and/or proliferation, increased proportion of apoptotic cells, and induced cell cycle arrest.

Taking advantage of the fact that cancer cells being more sensitive to the heat, hyperthermia has been explored and exploited for decades in cancer treatments [42, 43]. Hyperthermia injures or kills cancer cells by disrupting the integrity of cell membrane, cytoskeleton and the mitochondrial machinery that leads to cell necrosis, or activating apoptosis and cell cycle arrest [4–6, 44]. Hyperthermia in combination with radiotherapies or chemotherapies has shown more promising clinical efficacy in treating various cancers, as the combination therapy leads to accumulation of cell toxicity, as well as increased blood flow and drug delivery [14, 15, 17, 18].

Since temperatures in excess of 60°C have been shown to kill cancer cells [45], we exploited the conventional hyperthermia with a range from 42°C to 45°C in our study. Haghniaz et al. showed a minimum temperature of 45°C was required to decrease the viability of the melanoma cell line B16F [1]. Our study obtained consistent results by using temperatures lower than 45°C and showed these temperatures failed to eliminate B16 cells. It is notable that the cell viability curve of B16 cells in the hyperthermia group was not sustained flat but started to climb up from day 3 (Figure 1A), suggesting that the capability of hyperthermia in reducing the cell viability was attenuated.

Following hyperthermia HSP's are overexpressed, these HSPs exhibit chaperone functions to maintain the protein homeostasis, and cytoprotective machineries are switched on to assist the tumour cells to adapt the cellular and microenvironmental changes. Our results demonstrated HSPB1 expression could be strikingly upregulated by hyperthermia (Figure 1A, D; Figure 5E), and knock down of HSPB1 further reduced cell viability and proliferation in hyperthermic treated melanoma cells when compared with either single agent alone (Figure 2A-C, Figure 7A-B), providing evidence that silencing of thermosensitive HSPB1 combined with hyperthermia could increase the sensitivity of melanoma cells to each agent and improve the overall impact on cell survival or cell proliferation. Importantly, we have proved these findings in both mouse and human melanoma cells, indicating this combination strategy is not limitedly applicable on one particular cell line. It is also noteworthy that such a combination strategy displayed considerable cytotoxicity in A375 melanoma cell line which endogenously expressed high level of HSPB1 protein (Figure 7B), implying the potential clinical utility of this combination strategy in eliminating melanoma cells with endogenous or exogenously induced high expression of HSPB1.

We also showed that reduction of HSPB1 in both murine and human melanoma cells increased apoptosis and cell cycle changes. These changes were not unexpected given that the anti-apoptotic role of HSPB1 has been well studied. HSPB1 has been reported to trigger the apoptotic cascade directly through caspase 3 inhibition and indirectly through promotion of BAX phosphorylation and BAD inactivation using AKt kinase and IGF-1 pathways, respectively [33, 46]. Hyperthermia, on the other hand, has been shown to activate apoptosis through MAPK signaling or by accumulation of damaged proteins in a cell [47]. Therefore, it is not surprising that combining HSPB1 with hyperthermia resulted in additive apoptotic effects. A more detailed pathway analysis will be a feature of future studies by our group.

In addition, HSPB1 has been reported to promote cell progression from G0/G1 to S phase [48], and our data showed that silenced Hspb1 produced such a potent G0/G1 arrest that almost all cells were arrested at the G0/G1 checkpoint if Hspb1 was not active in the B16 cells. G2/M arrest is a feature of hyperthermia, however, cell cycle arrest features due to hyperthermia were masked by Hspb1 silencing. Despite Hspb1 silencing being the dominant mechanism in terms of cell cycle control, this result also highlighted the differing mechanisms of action of hyperthermia and Hspb1 silencing, and this bodes well in terms of reduced resistance to therapy.

Given the role of some heat shock proteins, such as HSP70, in immunosurveillance [29–31], HSP inhibitors may abrogate the anti-cancer immune responses at the same time as eliminating cancer cells. Therefore, it will be important to establish the immunoregulatory role of HSPB1, if any, in further investigation of this protein. In conclusion, we present evidence that HSPB1 silencing may improve cancer cell thermosensitivity when in combination with hyperthermia and have clinical potential in cancer treatments.

## MATERIALS AND METHODS

### Cell culture

Melanoma cell lines were were maintained in complete growth medium of Roswell Park Memorial Institute medium (RPMI, Life technologies) or Dulbecco's modified Eagle medium (DMEM, Life technologies) (RPMI for the murine B16 melanoma cell line and human melanoma cell line A375; DMEM for the human melanoma cell line MDA-MB-435S, supplemented with 10% fetal bovine serum (FBS, Gibco) at 37°C in a humidified incubator with 5% CO_2_.

### MTS assay

Cell viability was measured by using the MTS assay (Promega, Madison, WI) following the manufacturer's instructions. In brief, the cells were seeded in 96-well plates at a density of 5000 cells/well, and the cell viability was determined by measuring the absorbance at 490 nm at day 0, day 1, day 2, day3 and day 4 when the cell density reach 100% of confluence. All assays were completed in octuplicates and repeated three times.

### Heat stress

Cells were grown in culture plates and subjected to heat stress in a water bath. The bottom of culture plates was submerged in the water and incubated at 39°C, 43°C or 45°C for 30 minutes. Control cells were incubated in the water bath at 37°C for 30 minutes.

### siRNA transfection

Cells were seeded the day before transfection in antibiotics-free Medium. Lipofectamine™ RNAiMAX (Thermo Fisher scientific, USA) was used as siRNA transfection reagent according to the manufacturer's instructions. Cells grown in medium only and cells treated with Silencer^®^ Select negative control siRNA 1 were used as negative controls. A final concentration of 10 nmol of siRNA was used after optimization. The targeted sequences for Hspb1 siRNAs (mouse) were: Mm_Hspb1_1-TCCGGAGGAGCTCACAGTGAA and Mm_Hspb1_2-TCGGTGCTTCACCCGGAAATA (Qiagen). The targeted sequence for HSPB1 siRNAs (human) was: GCCGCCAAGUAAAGCCUUA (Ambion).

### Real time PCR (RT-PCR) analysis

RNA was extracted by miRNeasy mini kit (Qiagen, Germany). cDNA was synthesized from RNA by GoScript™ Reverse Transcription System (Promega, USA). Primers for mouse Hspb1 (Mm_Hspb1_1_SG) and the endogenous control B2m (Mm_B2m_2_SG) were purchased from Qiagen. Primers for human HSPB1 (F: 5′ CACGCAGTCCAACGAGATCA; R: 5′ AAAGAACACACAGGTGGCGG) and GAPDH (F: 5′ CCAGCAAGAGCACAAGAGGAAGAG; R: 5′ GTCTACATGGCAACTGTGAGGAG) were purchased from Invitrogen. RT-PCR was performed in 96-well plates by 7900HT Fast Real-Time PCR system (Thermo Fisher scientific, USA). Each reaction mixture is composed of 10μl GoTaq® qPCR Master Mix, 0.2μl CXR Reference Dye, 2μl primer, cDNA template (100ng) and additional Nuclease-free water to a final volume of 20μl. Ct values were calculated using RQ Manager Software (Thermo Fisher scientific, USA). Each assay was performed in triplicate and repeated three times.

### Immunofluorescence

Cells grown as monolayer were fixed with 4% paraformaldehyde for 20 minutes and permeabilized by 0.1% triton for 10 minutes. Cells were then blocked by 0.3% BSA for 1 hour, incubated with HSPB1 antibody (Abcam, China) at 4°C overnight, incubated with fluorescent conjugated secondary antibody in the dark for 2 hours before visualisation by confocal microscope. Nuclei were visualized by 4′, 6-diamidino-2-phenylindole (DAPI) staining.

### Western blot

Adherent cells were lysed by Radio-immune Precipitation (RIPA) buffer (Thermo Scientific), supplemented with a cocktail of protease inhibitors. Protein concentration in cell lysate was measured with the BCA Protein Assay Kit (Pierce, USA). 30μg of total protein from each sample were electrophoresed on a NuPAGE® 4-12% Bis Tris gel, and the proteins on the gel were transferred onto a PVDF membrane contained by iBlot® Gel Transfer Stacks (Invitrogen, Israel). The membranes were blocked with TBST with 5% non-fat milk for one hour at room temperature, incubated with HSPB1 antibody (Abcam, China) or ACTIN (Proteintech, China) overnight at 4°C, and incubated with a HRP-conjugated secondary antibody and detected by Clarity™ Western ECL Substrate (Bio-Rad, USA) on the following day.

### Flow cytometry

B16 cells were divided into six groups: untreated cells 37°C, siRNA negative 37°C, siHspb1-1 37°C, untreated cells 45°C, siRNA negative 45°C and siHspb1-1 45°C. For apoptosis and necrosis analysis, B16 cells were stained with the FITC Annexin V apoptosis detection kit (BD Biosciences, USA) in accordance with the manufacturer's instructions. In brief, the cells were trypsinized and washed with cold PBS, incubated with 5 μL of FITC Annexin V and 5μL of propidium iodide (PI) for 15 minutes at room temperature in the dark, and then analyzed within 1 hour. For cell cycle analysis, the cells were fixed with 70% cold ethanol overnight, and each sample was incubated with 1ml of PI (40 ug/ml) and 50 μl of RNase A (10 ug/ml) at 37°C for 20 minutes. Samples for apoptosis and cell cycle distribution were analyzed by BD LSRFortessa (BD Biosciences, USA), and the percentage of cells at G0/G1, S, or G2/M phase was analysed using ModFit software (Becton Dickinson, USA). Experiments were repeated three times.

### Colony formation

Six groups of B16 cells: untreated cells 37°C, siRNA negative 37°C, siHspb1-1 37°C, untreated cells 45°C, siRNA negative 45°C and siHspb1-1 45°C, were plated in 60 mm dishes (200 cells/dish) and cultured for 2 weeks to allow assessment of colony formation. The colonies were fixed with methanol and stained with 0.1% crystal violet before counting. Experiments were performed in triplicate.

### Statistical analysis

Statistical analysis, including Student's t test and one-way analysis of variance (ANOVA) were all performed by GraphPad Prism software, and a p value<0.05 was considered to be statistically significant.

## SUPPLEMENTARY MATERIAL FIGURES


