# Supplementary material for: HSPB1 deficiency sensitizes melanoma cells to hyperthermia induced cell death
Source: Oncotarget. 2016 Sep 8;7(41):67449–62. doi: 10.18632/oncotarget.11894 (PMC5341888; doi:10.18632/oncotarget.11894)
Supplement: Supplementary file 1 [file oncotarget-07-67449-s001.pdf]

HSPB1 deficiency sensitizes melanoma cells to hyperthermia induced cell death

SUPPLEMENTARY FIGURES

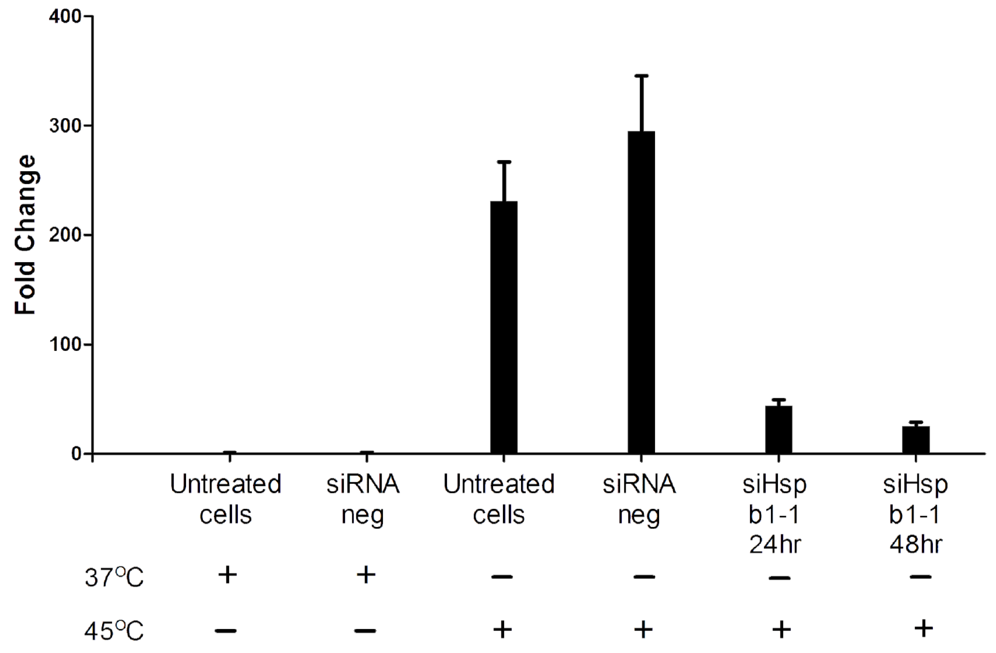

Supplementary Figure S1: mRNA expression of Hspb1 after siRNA transfection and/or hyperthermia (45°C) in B16 cell line. The mRNA expression level of Hspb1 was normalized against the endogenous control of B2m.

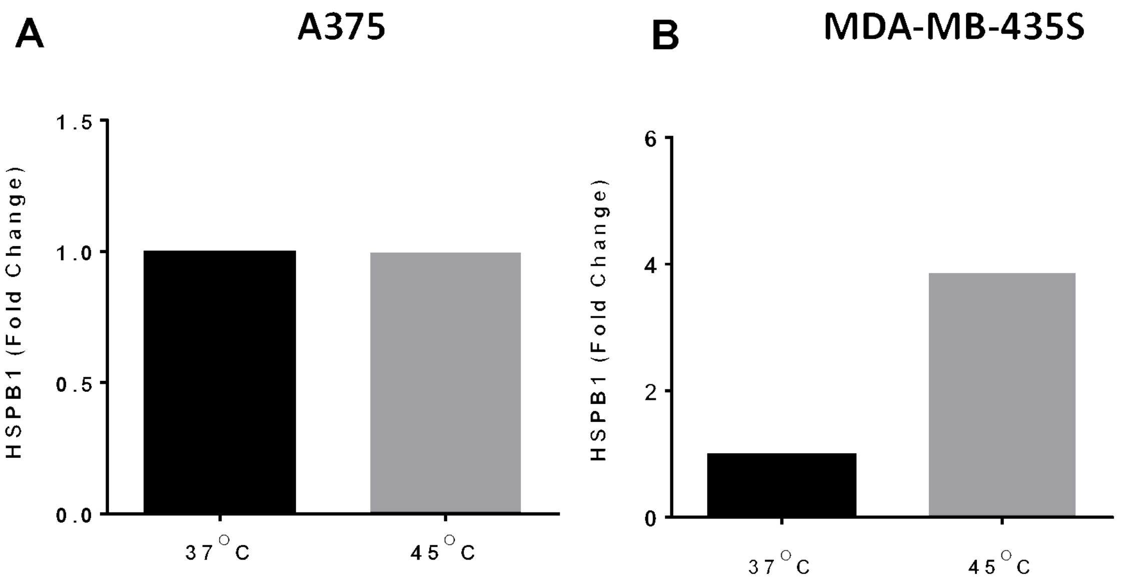

Supplementary Figure S2: Quantification of the HSPB1 protein expression relative to ACTIN in A375 cell line A. and MDA-MB-435S cell line B. using Image J software.

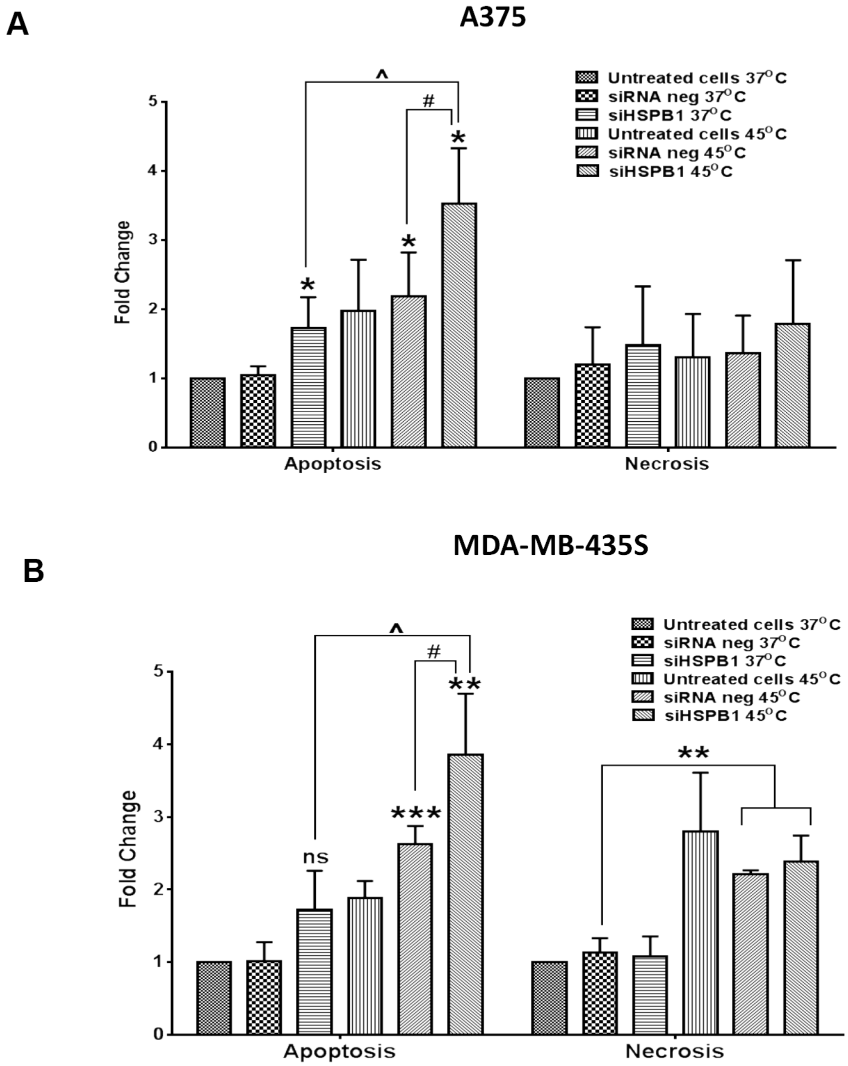

Supplementary Figure S3: The effect of hyperthermia (45°C) and HSPB1 knock down on apoptosis and necrosis in A375 A. and MDA-MB-435S B. cell lines analyzed by flow cytometry. Apoptosis and necrosis level of two cell lines were shown as fold change against siRNA negative group (37°C), and data shown were means from three independent experiments ± SD. * indicates a significant difference between each group and siRNA negative (37°C) (* $p<0.05$, by one way ANOVA); ^ siHSPB1 (45°C) vs siHSPB1 (37°C) ($p<0.05$, by student's t-test); # siHSPB1 (45°C) vs siRNA negative (45°C) ($p<0.05$, by student's t-test); ns, not significant.

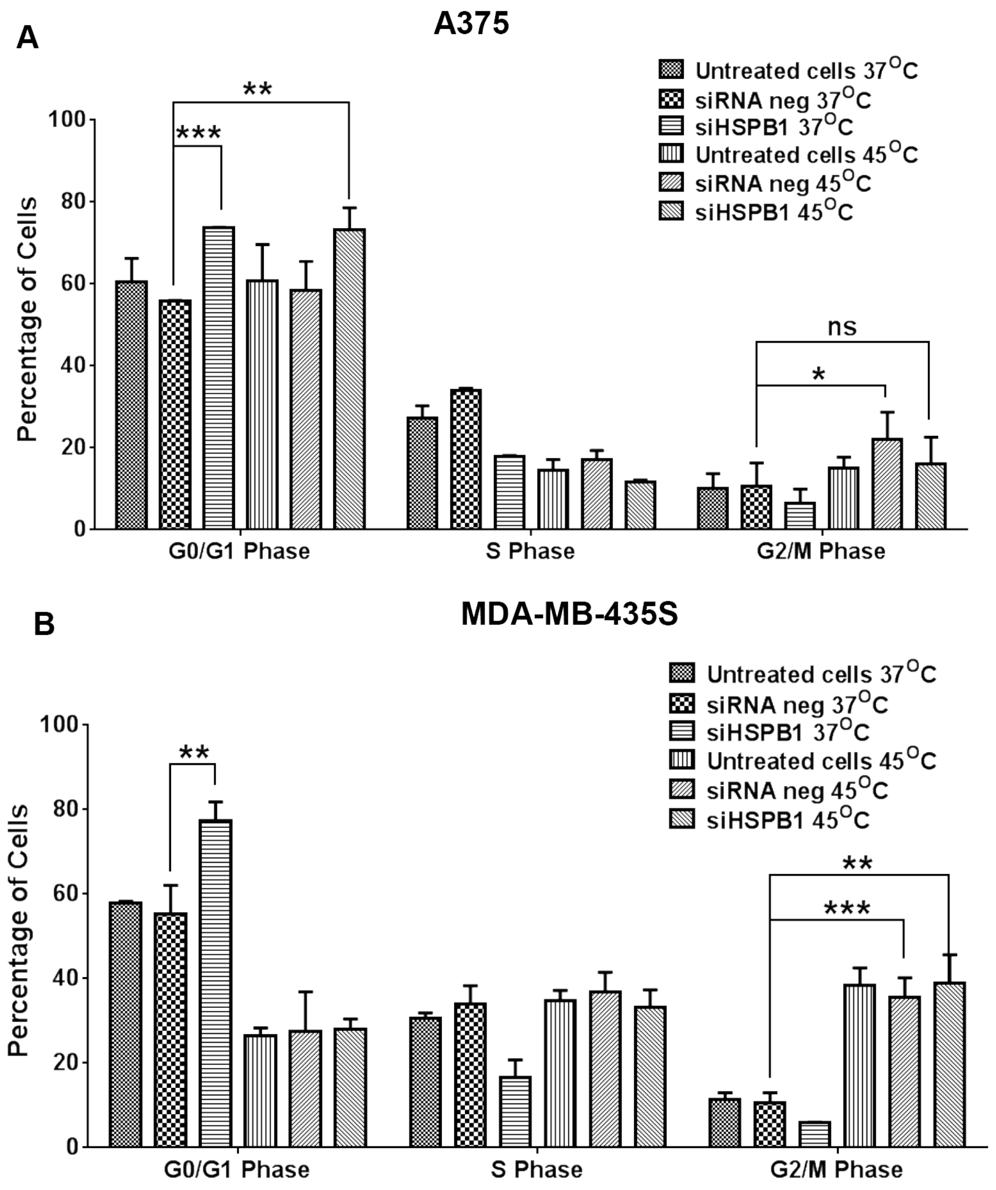

Supplementary Figure S4: Flow cytometric analysis of the effect of heat shock (45°C) and HSPB1 knock down on cell cycle regulation in A375 A. and MDA-MB-435S B. cell lines using PI staining. Graphs showed the mean values from three independent experiments ± SD. Statistical significance was calculated by Student's t-test: ns, not significant, * $p<0.05$, ** $p<0.01$, *** $p<0.001$.
